# T-cell activation of *Toxoplasma gondii* positive donors by maltodextrin nanoparticles formulated with killed Toxoplasma gondii

**DOI:** 10.1186/s12879-025-10656-5

**Published:** 2025-02-26

**Authors:** Monica Vargas-Montes, François Fasquelle, Nestor Ivan Cardona, Jorge Enrique Gómez-Marín, Didier Betbeder

**Affiliations:** 1 Center for Biomedical Research CIBM, University of Quindío, Armenia, Colombia; 2 Vaxinano, Loos, France; 3 Dentistry Faculty, University Antonio Nariño, Armenia, Colombia

**Keywords:** PBMC, Nanoparticles, *Toxoplasma gondii*, Antigen delivery, Interferon-gamma, Vaccine

## Abstract

Nasal vaccination with killed tachyzoites from *Toxoplasma gondii* loaded in lipidated maltodextrin nanoparticles (NPL/*T.gondii*) has been shown to induce protection against lethal *T.gondii* infection in mice, sheep and squirrel monkeys. This protection was related to a unique T-cell mediated response (Th1 and Th17). In immunocompetent humans *T.gondii* infection is generally asymptomatic but leads to the formation of cysts in several organs. These cysts may reactivate during episodes of immunosuppression, owing to a Th1 immune exhaustion, leading to numerous complications. The objectives of the current study were to assess whether NPL/*T. gondii* vaccine can stimulate a specific IFN-γ secretion on peripheral blood mononuclear cells (PBMC) of Toxo-positive donors, in order to potentially prevent reactivation of the parasite. By using enzyme-linked immunospot (ELISpot) assays, the frequency of IFN-γ producing cells was quantified after 72 h of stimulation. This stimulation was specific of the Toxo + donors’ memory T-cells. The uptake of this formulation by PBMCs was evaluated using flow cytometry, more particularly in T lymphocytes (CD3 + CD4 + and CD3 + CD8 +), B lymphocytes (CD3- CD19 +) and natural killer cells (CD3- CD56 +). Surprisingly, a lower antigen uptake was observed in all cell populations from Toxo + donors compared with the seronegative ones. Further in vitro analysis revealed that the early specific IFN-γ secretion by stimulated memory cells from Toxo-positive donors inhibited the endocytosis mechanism, therefore lowering their own vaccine uptake. These results suggest that NPL/*T.gondii* formulation can specifically trigger the existing Th1 immune response in positive donors, reinforcing their ability to control infection and reactivation.

**Supplementary Information:**

The online version contains supplementary material available at 10.1186/s12879-025-10656-5.

## Introduction

Toxoplasmosis is a disease caused by the infection with the protozoan parasite *Toxoplasma gondii*, mainly via the ingestion of contaminated food or water. When seronegative women become infected during pregnancy, it can dramatically affect the development of the fetus, but most immunocompetent individuals show very few symptoms upon infection. The parasite then remains latent in the body for years in a cyst form, mainly in the brain and muscles. Cysts can reactivate during episodes of immunodepression or immuno-suppression (e.g., during illness or owing to medication), leading to severe local damage. Reactivation of any cysts formed in the eye can lead to uveitis or retinochoroiditis, and even blindness if left untreated [1].

Despite the absence of symptoms, the first infection triggers the innate immunity leading to the establishment of an adaptive Th1/Th2 immune response evident in serological analysis. The parasite evades innate immune responses through the modulation of the host signaling pathways. This modulation induces the survival of infected cells and leads to the formation of a replicative niche, overcoming the establishment of an adaptative immune response [2].

Subsequent *T.gondii* reactivation is normally attenuated by the naturally developed Th1 memory immune response in which lymphocytes producing IFN-γ (CD3 + CD4 + and CD3 + CD8 +) play a major role [3, 4]. Their specific secretion of IFN-γ in proximity to infected macrophages indeed hinders intracellular parasite growth by triggering the production of both cytolytic inducible nitric oxide synthase (iNOS) and indoleamine 2,3-dioxygenase (IDO), an enzyme involved in the tryptophan catabolism which is crucial for the parasite’s survival. Moreover, cytotoxic CD3 + CD8 + can kill both infected cells and extracellular parasites. Importantly, this Th1-oriented immune response is supported by dendritic and natural killer (NK) cells, and sustained by the secretion of co-stimulatory cytokines such as TNF, IL-1β, IL-2 or IL-12. However, recent studies have suggested that parasite reactivation occurs in conjunction with an exhaustion of memory lymphocytes [5–7]. Therefore, the administration of an immunotherapy in infected donors could boost the exhausted memory Th1 response and help block the parasite reactivation.

To date, although many different approaches have been found to be successful in mice models, including live attenuated or inactivated parasites, DNA, proteins, peptides and vector-based vaccines, no prophylactic vaccines against toxoplasmosis are available for humans owing to several difficulties encountered during their clinical translation [8, 9]. In addition, while vaccines based on killed organisms, total lysates and excretion/secretion products have been described as effective approaches, these approaches only aim to prevent *T.gondii* infection without preventing parasite reactivation. Furthermore, the success of these vaccines strongly depends on the immunostimulatory properties of adjuvants, with the attendant risk of side effects [10].

Regarding these considerations, nanoparticles (NP) are frequently considered as delivery systems to enhance antigens’ immunogenicity, although they often require the use of adjuvants. They contribute to the protection of the antigen integrity against enzymatic degradation, prolonging the local or systemic residence time, and enhance antigen delivery to innate immune cells [11, 12]. Further, NPs can also have an immunomodulatory effect and can act as powerful adjuvants, reducing the effective-dose threshold, and consequently the risk of toxicity and side effects [13, 14].

Lipidated maltodextrin-based NPs (NPL) are delivery systems designed for nasal immunization, since they are able to encapsulate large amounts of antigens and to be highly endocytosed by mucosal cells [15]. Due to their anionic phospholipid core, they are able to cross the mucus layer and reach the underlying epithelial cells, thus increasing the antigen residence time after nasal administration [16, 17]. Additionally, NPL are safe as they are not immunomodulators by themselves and do not induce any inflammation, but they remain an efficient delivery system [18, 19]. Nasal immunization with NPL loaded with antigens is thus a promising approach to improve vaccination against infectious diseases.

Recently, NPL loaded with killed *Toxoplasma gondii* have been used as a nasal vaccine in different animal species including non-human primates [20]. The formulation induced a specific Th1/Th17 immune response after vaccination, which was associated with high protection against latent and congenital toxoplasmosis, as evidenced by protection against lethal challenge and abortion when the infection occurs during pregnancy [21, 22]. This vaccine has been used with success on New World monkeys in zoological parks to prevent against lethal *T.gondii* infection [23].

The objectives of the current study were to assess whether NPL/*T. gondii* vaccine can stimulate a specific Th1 memory immune response in infected blood donors, in order to boost their existing immune response and prevent parasite reactivation. The interactions between the vaccine and peripheral blood mononuclear cells (PBMC) were also assessed by measuring the antigen uptake by each cell subtype, by flow cytometry.

## Materials and methods

### Material

Histopaque-1077, Dulbecco's phosphate buffered saline (DPBS), Roswell Park Memorial Institute medium (RPMI 1640), Dulbecco's Modified Eagle Medium (DMEM), Fetal calf serum (FCS), Dimethylsulfoxide (DMSO), Penicillin, Streptomycin, L-glutamine, 2-mercaptoethanol,Trypan blue 0.4% and flow cytometry staining buffer were all purchased from ThermoFisher (France). Fluorescein isothiocyanate (FITC), Phorbol 12-myristate 13-acetate (PMA) and ionomycin calcium salt were purchased from Sigma-Aldrich (France). Human anti-CD3-Alexa Fluor 700, anti-CD4-PerCP-Cyanine 5.5, anti-CD8-PE.Cyanine7, anti-CD56-APC, anti-CD19-APC-eFluor 780, flow cytometry staining buffer and Fc receptor blocking solution (Human TruStain FcX) were purchased from Biolegend (France). Toxoplasma serological test was purchased from LD Bio (France). ELISPOT plates were purchased from Mabtech (Sweden). Vero cells were obtained from ATCC (CCL-81), as well as *T.gondii* tachyzoites (ATCC 50174), and stored in liquid nitrogen until use.

### Human samples—Ethics approval and consent to participate

The study was conducted in accordance with the Declaration of Helsinki. Heparinized blood samples were spared from blood donation nonrelated to the study, of anonymous and healthy volunteers, regardless of their previous *Toxoplasma* infection states, and collected by the Etablissement Français du Sang (EFS, convention PLER-UPR/2020/002). Despite not being recruited specifically for the study, all donors gave their written consent that the blood could also be used for research purpose. Blood samples were donated by the EFS for the project with the approval and in accordance with the French Research Agency recommendations, for specific research use only (Article L1221-8–1, French Public Health Code). All blood samples were processed within 24 h of sampling.

### PBMCs isolation

The blood samples were collected in heparinized blood tubes, and initially subjected to a serological IgG-IgM rapid diagnostic test (Toxoplasma ICT IgG-IgM, LDBio, France) to determine the seropositive / seronegative status for the infection. The PBMC were then isolated by Histopaque-1077 density gradient centrifugation. Briefly, 10 mL of blood were diluted with 10 mL of DPBS at room temperature then slowly layered onto 20 mL of Histopaque in 50 mL conical tubes and centrifuged at 400 × g for 30 min. The mononuclear cell fraction in the opaque interface was then collected and washed twice in DPBS at 300 × g for 10 min. The cells were then counted and resuspended in freezing media (RPMI with 10% FBS, 50 μM 2-Mercaptoethanol, 1% Pen/Strep and 10% DMSO) and cryopreserved at −80 °C for short term storage until use.

### Parasite culture and inactivation

Vero cells were first seeded in T75cm^2^ flasks with 10 mL supplemented RPMI 1640 (10% heat-inactivated FCS, 1% Penicillin/Streptomycin) until they reach 80% confluency, in a cell incubator at 37 °C and with 5% CO_2_. Tachyzoites (*T.gondii* type I) were thawed at 37 °C in a water bath, and 10^8^ parasites/mL were seeded in the Vero flasks. Infected Vero cells were then subcultured in T225cm^2^ flasks. When the Vero cell full lysis was reached, the supernatant was harvested. Tachyzoites were then purified from the cell debris by centrifugation and washed 3 times in DPBS by centrifugation.

After harvesting, the parasite suspension was concentrated at 10^7^ parasites/mL, and they were inactivated by 6 consecutive freeze/thaw cycles at −80 °C. The total parasite inactivation was confirmed by Vero cell infection. The antigen dose was expressed as total protein content, calculated by microBCA assay (Pierce, France).

### Vaccine formulation and *T. gondii* labeling with FITC

The vaccine formulation was prepared in water by mixing premade NPL with killed *T.gondii*, at a 1:1 weight ratio and without any adjuvant, as previously described [24]. The antigen association was evaluated by running the formulation into a non-denaturing polyacrylamide gel electrophoresis (Native PAGE); the percentage of association was quantified using ImageJ, by comparing the lanes containing free parasite antigens with those containing the NPL formulation, using the following calculus:

$$\% association= 100-(\frac{AUC of the formulatio{n}{\prime}s lanes }{AUC of the free antige{n}{\prime}s lanes}*100)$$

The size and surface charge of the formulation were characterized by dynamic light scattering (DLS) and electrophoretic light scattering (ELS) respectively (Nanosizer Nano ZS, Malvern, France).

For the antigen delivery study, killed *T.gondii* were labeled with FITC (*T.gondii*-FITC) before loading in NPL. Briefly, the antigens were mixed with 1% (w/w) of FITC in sodium carbonate buffer (pH 8.3). The mixture was then stirred with magnetic stirring at room temperature for 2 h. The *T.gondii*-FITC was then purified from the unbound dye by dialysis in 10 kDa dialysis cassettes (ThermoFisher, France). Antigen labelling was confirmed by fluorimetry (Fluoroskan Ascent, ThermoFisher, France) and the final protein concentration was measured using a microBCA assay, before *T.gondii*-FITC was finally mixed with premade NPL to obtain the NPL/*T.gondii*-FITC formulation.

### Enzyme-linked immunospot (ELISpot) assay

An ELISpot assay was used to quantify the frequency of IFN-γ secretion by PBMC stimulated with NPL/*T.gondii*. Frozen PBMC were thawed in a 37 °C-water bath then seeded in 96-well plates (Fisher Scientific, France) at 10^7^ cells/mL in 100µL. They were stimulated with either complete RPMI as a negative control, 10 ng/mL of PMA and 1 μg/mL of Ionomycin as a positive control, or with 10 µg/mL of NPL/*T.gondii* formulation for 48 h at 37 °C and 5% CO2. Then, for each condition, 2 × 10^5^ cells were carefully harvested and seeded in 96-well precoated IFN-γ ELISpot plate, in 100µL media. The plate was incubated at 37 °C with 5% CO_2_ for 24 h. IFN-γ-representative spots were then revealed as described by the manufacturers and counted in an ELISpot reader (Astor, Mabtech, Sweden). Antigen-specific responses were expressed as spot-forming units (SFUs) per 2 × 10^5^ PBMCs and normalized by subtraction of spots from negative control wells.

### Uptake of NPL/*T.gondii*-FITC by human PBMC

**Fig. 1:**
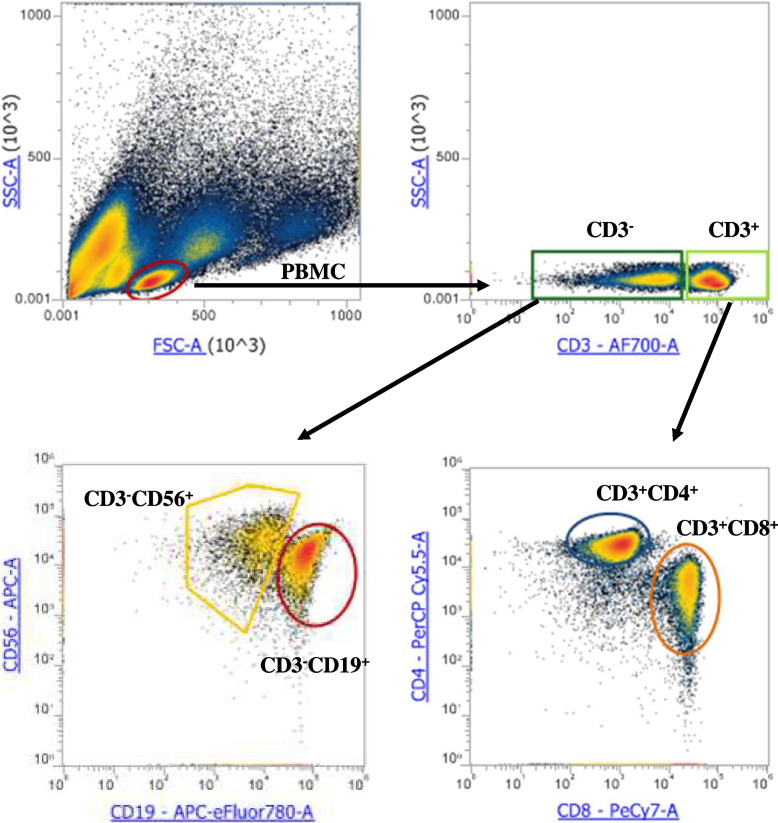
Flow cytometry gating strategy to analyze PBMCs subsets, namely T lymphocytes (CD3^+^CD4^+^ and CD3^+^CD8^+^), B lymphocytes (CD3^−^ CD19^+^) and natural killer cells (CD3^−^ CD56^+^). The NPL/*T.gondii*-FITC uptake was assessed in terms of percentage of positive cells and of Median fluorescence intensity (MFI), in comparison with untreated cells

Frozen PBMC were thawed in a 37 °C-water bath. They were seeded at 10^7^ cells/mL in 96-well plates and in 100µL media. They were incubated with complete RPMI, or with NPL/*T.gondii*-FITC at 10 µg/mL, in a cell incubator at 37 °C with 5% CO_2_. After 24 h incubation, PBMC were harvested, washed with 1 mL of DPBS at 4 °C and centrifuged at 350 × g for 10 min. Cell pellets were resuspended in flow cytometry staining buffer and incubated with a FcR blocking reagent for 5 min at 4 °C. Cells were incubated for 20 min at 4 °C with the following pre-titrated antibodies: human anti-CD3-Alexa Fluor 700, anti-CD4-PerCP-Cyanine 5.5, anti-CD8-PE.Cyanine7, anti-CD56-APC, and anti-CD19-APC-eFluor 780. Then the cells were diluted in flow cytometry staining buffer and analyzed with the Attune NxT Flow Cytometer (ThermoFisher Scientific, France). In all cases at least 20,000 PBMCs were acquired according to the gating strategy (Fig. 1). The formulation uptake by CD3^+^CD4^+^ and CD3^+^CD8^+^ T lymphocytes, CD3^−^CD19^+^ B lymphocytes, and CD3^−^CD56 + natural killer cells were analyzed in terms of percentage of positive cells and of Median fluorescence intensity (MFI) for the FITC channel. Data were analyzed on the Attune NxT software.

### Influence of IFN-γ on NPL/*T.gondii* uptake by PBMCs

Frozen PBMC from seronegative donors were thawed in a 37 °C-water bath and seeded at 10^7^ cells/mL in 96-well plates and in 100µL media. They were incubated with 20 ng/mL IFN-γ for 1 h in a cell incubator at 37 °C with 5% CO_2_. Then complete RPMI, or 10 µg/mL NPL/*T.gondii*-FITC was added for 4 h. The cells were finally harvested, labeled and prepared for flow cytometry, as described above.

## Results

### Characterization of the NPL/*T.gondii* formulation

**Fig. 2:**
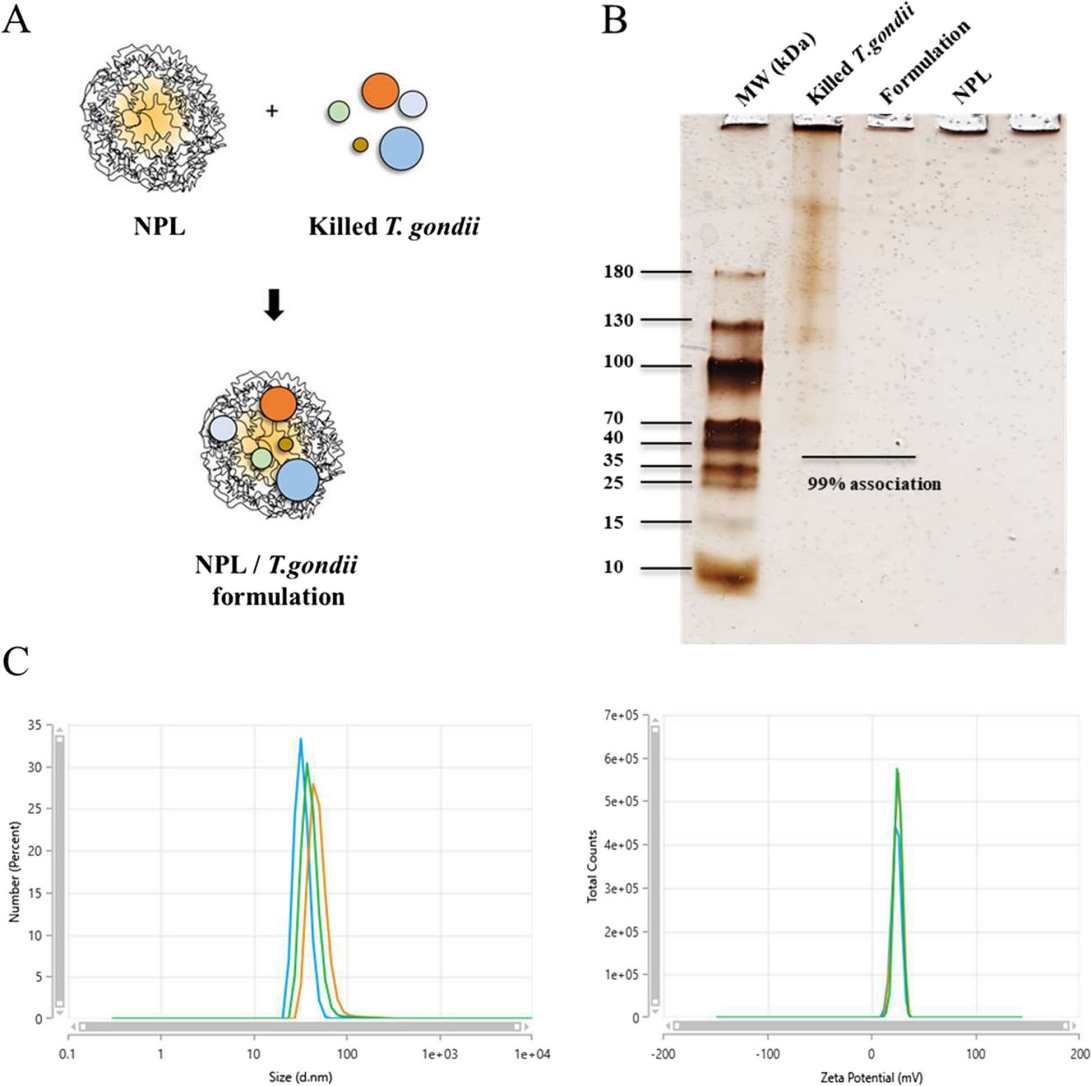
Physicochemical characterization of the NPL/*T.gondii* vaccine formulation. **A**: Schematical representation of the NPL/*T.gondii* formulation. **B**: Native-PAGE of the NPL/*T.gondii* formulation compared with free *T.gondii* and to empty NPL. The % of encapsulation is calculated by Image J software. **C**: Graphical representation of the size (left) and surface charge (right) triplicate analysis, by DLS and ELS respectively

The vaccine formulation was made by mixing NPL and killed *T.gondii* in water (Fig. 2A). Native PAGE analysis revealed an intense smear in the killed *T.gondii* well, while almost no protein migrated in the gel from the formulation (Fig. 2B). The quantification of the association revealed that 99% of the proteins were associated with the NPL. Moreover, size and zeta-potential analysis revealed that the formulation had a 42 nm diameter, with a + 24 mV surface charge (Fig. 2C) while free NPL had a 25 nm diameter and a + 33 mV surface charge (see Additional file 1). This confirmed that the antigens were mostly loaded inside the NPL, with only a small proportion associated on their surface, thus slightly increasing the apparent size, and lowering the surface charge.

### PBMC stimulation by NPL/T.gondii formulation

**Fig. 3:**
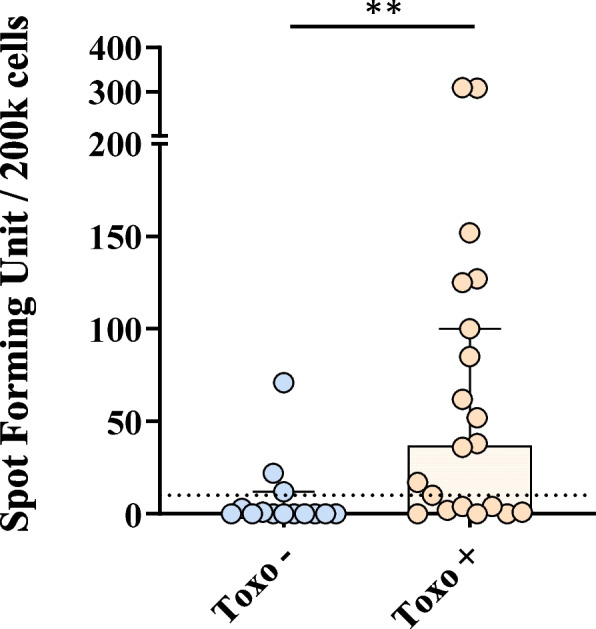
Evaluation of the PBMC stimulation by the NPL**/**T.gondii vaccine formulation*.* Spot forming units (SFU) of IFN-γ-producing cells detected by ELISpot in PBMC from 14 *Toxoplasma* seronegative (Toxo -) and 20 *Toxoplasma* seropositive (Toxo +) individuals incubated with 10 µg/mL of the formulation NPL**/**T.gondii. Dashed line represents the arbitrary positive threshold = 10 SFU. Data are presented as median ± 95% IC and statistical comparisons were made by Mann–Whitney comparisons test, ** *p* < 0.01

The NPL/*T.gondii* formulation was incubated with PBMC of seronegative (Toxo -) and seropositive (Toxo +) blood donors (Fig. 3). The production of IFN-γ was assessed by ELISpot. A significantly greater number of IFN-γ spots were observed from Toxo + individuals after stimulation (median = 37 spots) compared with Toxo – donors (median = 0 spot, *p* = 0.0012). This confirmed that the formulation can specifically stimulate the memory response on infected donors.

### Uptake of NPL/*T.gondii* by PBMC

To understand the interactions between the vaccine formulation and PBMC, the delivery of NPL/*T.gondii*-FITC was evaluated on PBMCs subsets by flow cytometry. It is noteworthy that the frequencies of PBMC subsets (CD3^+^CD4^+^, CD3^+^CD8^+^, CD3^−^ CD56^+^, and CD3^−^ CD19^+^) were not statistically different between Toxo – and Toxo + donors (see Additional file 2).

**Fig. 4:**
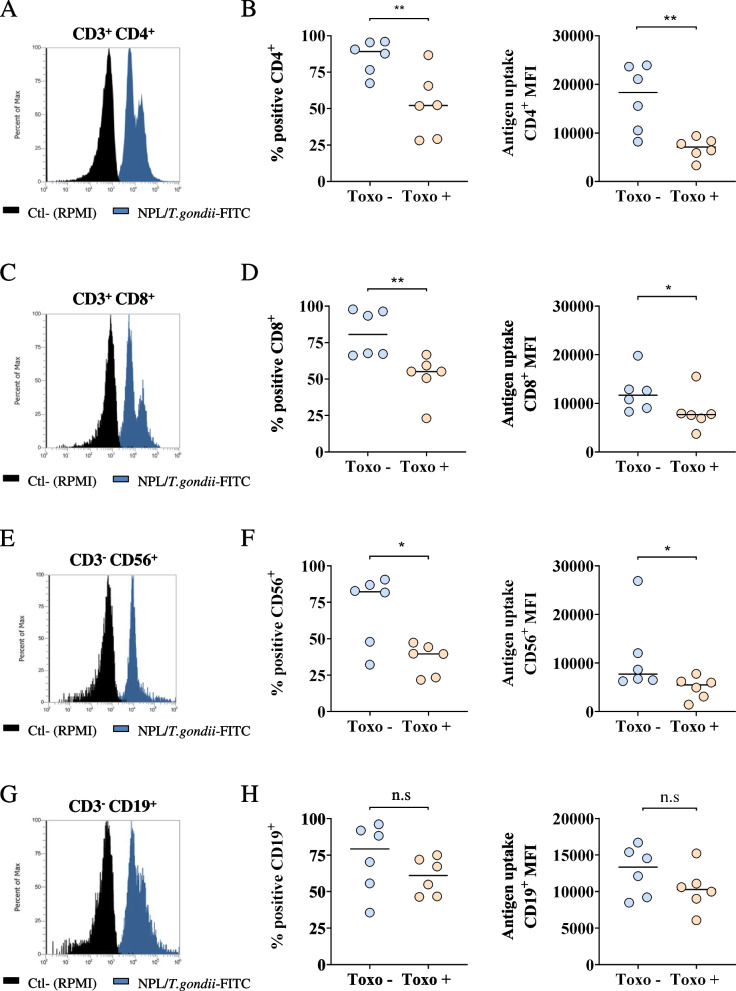
Analysis of the differential uptake of NPL/*T.gondii*-FITC in CD3^+^CD4^+^, CD3^+^CD8^+^, CD3^−^ CD56^+^, and CD3^−^ CD19^+^ PBMC subsets from 6 Toxo + and 6 Toxo – blood donors. Histograms represent CD3^+^CD4^+^ (**A**), CD3^+^CD8^+^ (**C**), CD3^−^ CD56^+^ (**E**), and CD3^−^ CD19^+^ (**G**) PBMC subsets, either untreated (black) or incubated with the NPL/*T.gondii* formulation (blue). Graphics represent the percentage of positive cells (left) and the Median Fluorescence Intensity (MFI, right) of CD3^+^CD4^+^ (**B**), CD3^+^CD8^+^ (**D**), CD3^−^ CD56^+^ (**F**), and CD3^−^ CD19.^+^ (**H**) PBMC subsets. Data are presented as median and statistical comparisons were made by Mann–Whitney comparisons test, * *p* < 0.05, ** *p* < 0.01

Surprisingly the antigen delivery was significantly greater in cell subsets from Toxo – donors compared with Toxo + (Fig. 4): for CD3^+^CD4^+^ cells, the vaccine was delivered in 89% of the cells from Toxo – donors, with an MFI of 18,312 units, and in 52% of the cells of Toxo + donors (*p* < 0.01), with an MFI of 7075 units (*p* < 0.01); regarding CD3^+^CD8^+^ cells, the vaccine was delivered in 81% of the cells from Toxo – donors, with an MFI of 11,698 units, and in 55% of the cells form Toxo + donors (p < 0.01), with an MFI of 7676 units (p < 0.05); for CD3^−^CD56^+^ cells, the vaccine was delivered in 79% of the Toxo – donors’ cells, with an MFI of 13,329 units, and in 61% of the Toxo + donors’ cells (p < 0.05), with an MFI of 10,288 units (*p* < 0.05). Moreover, a small, non-significant decrease was observed for CD3^−^CD19^+^ cells between the two populations. The stimulation of T-cells of Toxo + donors induced the production of IFN-γ and this production might have had an effect on the uptake of antigen by the cells.

### Impact of IFN-γ on NPL/*T.gondii* uptake by PBMC

To elucidate the impact of IFN-γ secretion on NPL/*T.gondii* uptake, PBMC from Toxo – blood donors were incubated with the fluorescently labelled vaccine formulation in presence of exogenous IFN-γ.

**Fig. 5:**
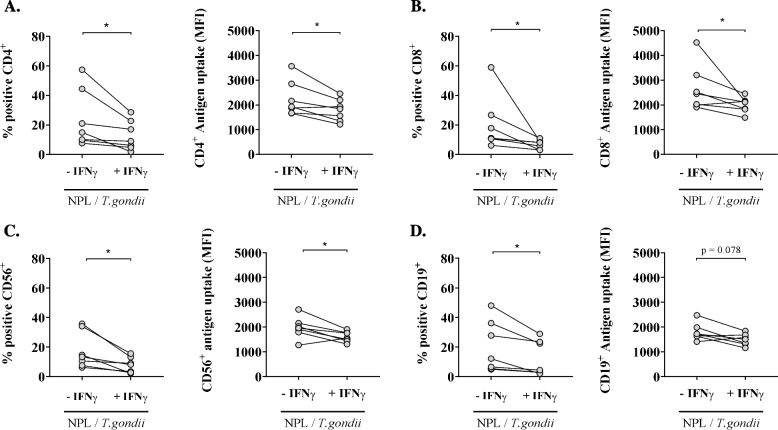
Analysis of the impact of IFN-γ on the NPL/*T.gondii*-FITC uptake in PBMC subsets from 7 Toxo – blood donors. Graphics represent the percentage of positive cells (left) and the Median Fluorescence Intensity (MFI, right) of CD3^+^CD4^+^ (**A**), CD3^+^CD8^+^ (**B**), CD3^−^ CD56^+^ (**C**), and CD3^−^ CD19.^+^ (**D**) PBMC subsets, in absence and presence of IFN-γ. Data were analyzed by Wilcoxon test. *p < 0.05

Interestingly, the delivery was significantly lower when PBMC were preincubated with IFN-γ (Fig. 5): for CD3^+^CD4^+^ cells, the vaccine was delivered in 23.6% of the cells without IFN- γ, with an MFI of 2245 units, and in 12.88% when IFN-γ was added (*p* < 0.05), with an MFI of 1794 units (*p* < 0.05); for CD3^+^CD8^+^ cells, the vaccine was delivered in 20.35% of the cells without IFN-γ, with an MFI of 2664 units, and in 6.89% in presence of IFN-γ (*p* < 0.01), with an MFI of 2003 units (*p* < 0.05); for CD3^−^CD56^+^ cells, the vaccine was delivered in 17.42% of the cells without IFN-γ, with an MFI of 1971 units, and in 7.73% of the cells with IFN-γ (*p* < 0.05), with an MFI of 1603 units (*p* < 0.05); for CD3^−^CD56^+^ cells, the vaccine was delivered in 17.42% of the cells without IFN-γ, with an MFI of 1971 units, and in 7.73% of the cells with IFN-γ (*p* < 0.05), with an MFI of 1603 units (*p* < 0.05); finally, for CD3^−^CD19^+^ cells, the vaccine was delivered in 20.08% of the cells without IFN-γ, with an MFI of 1794 units, and in 12.37% of the cells with IFN-γ (*p* < 0.05), with an MFI of 1488 units (*p* = 0.078).

These results confirmed that IFN-γ inhibits the vaccine uptake by PBMCs.

Altogether, these observations suggest that the NPL/*T.gondii* formulation can specifically trigger a memory immune response in PBMC from Toxo + blood donors, leading to the secretion of IFN-γ which will subsequently inhibit the endocytosis of the NPL formulation.

## Discussion

*Toxoplasma gondii* infections are generally asymptomatic for immunocompetent donors, and the parasite can remain latent in the body for decades. The parasitic infection mainly relies on evasion of the host immune system through inhibition of the innate immune signaling pathways, allowing it to survive hidden within infected cells [2]. For instance, *T.gondii* secretes effector proteins from rhoptries and dense granules within infected cells, which inhibit the activation of IFN-γ genes and consequently the mechanism of clearance [25].

Recent studies have described that the parasite reactivation in chronically infected host is a consequence of CD8 T-cell dysfunction, caused both by CD4 T-cell exhaustion and by NK negative regulation [6, 26]. In parallel, specific CD8 T-cells are able to clear cysts from immunodeficient, infected animals [27]. All these findings suggest that a stimulation of the adaptive immune system in chronically infected donor through immunization could prevent parasite reactivation, and potentially clear cyst from infected tissues.

We have shown that NPL loaded with killed *T.gondii* antigens is an efficient vaccine to prevent *T.gondii* infection and vertical transmission in mice and sheep, and most recently reported the induction of a protective immune response against lethal toxoplasmosis in non-human primates [21–23]. In light of these promising results, we sought to determine if the vaccine could also stimulate the extant adaptive response in Toxo + humans, with the aim of preventing the T-cell exhaustion and therefore the parasite reactivation.

Therefore, the NPL/*T.gondii* formulation’s ability to specifically trigger the memory immune response in infected donors in comparison to non-infected donors was assessed in vitro by incubating PBMC from asymptomatic Toxo + or Toxo- blood donors with the NPL/*T.gondii* formulation and measuring the subsequent IFN-γ secretion. The ELISpot analysis revealed a significant secretion of IFN-γ from Toxo + compared with Toxo- donors (*p* = 0.0012), confirming the specific stimulation of memory cells. While the recognition between an epitope and the T-cell receptor (TCR) is highly specific, unspecific stimulations might also occur through an innate recognition between pathogen-associated molecular pattern (PAMP) and toll-like receptors (TLR) [28]. Here, only 3/14 Toxo- donors were activated and secreted IFN-γ, suggesting relatively few non-specific recognitions. A deeper evaluation of the serological data (such as IgM/IgG positivity) to establish whether the donors are in an active or chronic phase of infection, could also give insights on the establishment of the adaptive immune response after the infection.

The NPL are an inert delivery system and don’t activate the immune system by themselves. Instead, antigen delivery by NPL is the only route of cell stimulation and sole cause of subsequent immune activation [19]. The difference in PBMC activation observed between the two populations by ELISpot must, then, be due to a better antigen delivery to Toxo + donors’ cells than to Toxo- donors’ cells, likely through specific receptor recognition.

To confirm this hypothesis, the delivery of fluorescent *T.gondii*-FITC to PBMC subpopulations was measured by flow cytometry. Surprisingly the delivery was in fact significantly greater in CD4^+^ and CD8^+^ T-cells and NK cells from Toxo- donors. Hence, triggering of the immune response is not directly linked solely to the amount of antigen delivered, and the lower delivery observed for Toxo + donors suggests the existence of a retro-control mechanism linked to the antigen recognition. The addition of T cell memory markers (such as CD45RO/RA, CD127, CD62L or CD44) would bring deeper information on the implication of memory cells in this recognition and activation.

The recognition between an epitope and the TCR from CD4^+^ and CD8^+^ T-cells is a rapid phenomenon that occurs within minutes [29]. This recognition leads to the internalization of the TCR, and to numerous intracellular processes linked to both the protection from an intracellular infection, and to the elimination of the pathogen. Among these processes, the secretion of IFN-γ helps defend infected macrophages by inhibiting parasite growth through production of inducible nitric oxide synthase (iNOS) and indoleamine 2,3-dioxygense (IDO), an enzyme which is essential for the parasite survival [3]. Moreover, IFN-γ is also known to inhibit endocytosis as a defense mechanism against infections. Gomez-Marin and colleagues found that the addition of IFN-γ to cultured, infected THP-1 macrophages reduced the number of parasitized cells, but did not alter intracellular multiplication of the parasite [30, 31]. They also reported that IFN-γ prevents the parasite from invading the cell by suppressing production and activity of secretory phospholipases-2 (PLA2), both in the parasite and the host cells (infected and non-infected). Thus, the authors concluded that IFN-γ blocks the activities of both THP-1 and parasite-secreted PLA2 and cytoplasmic PLA2 in membrane fractions, resulting in protection against active invasion by *T. gondii*. Our data would appear to confirm their observations.

The IFN-γ receptor is expressed on the surface of all mammal cells. Therefore, the early secretion of IFN-γ by PBMC from Toxo + donors when incubated with the NPL/*T.gondii* formulation could inhibit the subsequent formulation endocytosis. To investigate this hypothesis, uptake of NPL/*T.gondii* formulation was assessed on PBMC from Toxo- donors after a short incubation with exogeneous IFN-γ, and antigen delivery was significantly lower for every PBMC subtype.

## Conclusion

The NPL/*T.gondii* vaccine formulation described here can trigger the existing Th1 adaptive immunity in Toxo + individuals through a specific recognition, confirmed by PBMC activation. This observation opens the possibility of using these formulations on PBMC from different patients with toxoplasmosis-related complication (chorioretinitis, encephalitis and congenital infection) to confirm its ability to boost preexisting immunity of infected donors and prevent reactivation of the parasite.

## Supplementary Information

Additional file 1. Characterization of sizeand surface chargeof empty NPL before the antigen encapsulation

Additional file 2. Frequencies of PBMC subsetsbetween Toxo – and Toxo + donors, measured by flow cytometry.

## Data Availability

The datasupporting the findings of the study are hosted in a public depository (OSF) and fully available using the following DOI : 10.17605/OSF.IO/4UT9Y.

## References

[1] Kalogeropoulos D, Sakkas H, Mohammed B, Vartholomatos G, Malamos K, Sreekantam S, et al. Ocular toxoplasmosis: a review of the current diagnostic and therapeutic approaches. Int Ophthalmol. 2022;42(1):295–321. 34370174 10.1007/s10792-021-01994-9 PMC8351587

[2] Lima TS, Lodoen MB. Mechanisms of human innate immune evasion by toxoplasma gondii. Front Cell Infect Microbiol. 2019;9:103. 31041194 10.3389/fcimb.2019.00103 PMC6476913

[3] Sturge CR, Yarovinsky F. Complex Immune Cell Interplay in the Gamma Interferon Response during Toxoplasma gondii Infection. Infect Immuny. 2014;82(8):3090–7 Andrews-Polymenis HL, editor. 10.1128/IAI.01722-14 PMC4136216 24866795

[4] Sasai M, Pradipta A, Yamamoto M. Host immune responses to *Toxoplasma gondii*. Int Immunol. 2018;30(3):113–9. 29408976 10.1093/intimm/dxy004

[5] García-López LL, Vargas-Montes M, Osorio-Méndez JF, Cardona N, Hernández De Los Ríos A, Toro-Acevedo CA, et al. CD8+ T–cell Exhaustion Phenotype in Human Asymptomatic and Ocular Toxoplasmosis. Ocular Immunology and Inflammation. 2023;1–10. 10.1080/09273948.2023.2217906 37315178

[6] Khan IA, Hwang S, Moretto M. Toxoplasma gondii: CD8 T cells cry for CD4 help. Front Cell Infect Microbiol. 2019;9:136. 31119107 10.3389/fcimb.2019.00136 PMC6504686

[7] Khan IA, Moretto M. Immune responses to Toxoplasma gondii. Curr Opin Immunol. 2022;77:102226. 35785567 10.1016/j.coi.2022.102226

[8] Wang JL, Zhang NZ, Li TT, He JJ, Elsheikha HM, Zhu XQ. Advances in the development of anti-toxoplasma gondii vaccines: challenges, opportunities, and perspectives. Trends Parasitol. 2019;35(3):239–53. 30718083 10.1016/j.pt.2019.01.005

[9] Zhang Y, Li D, Lu S, Zheng B. Toxoplasmosis vaccines: what we have and where to go? npj Vaccines. 2022;7(1):131. 36310233 10.1038/s41541-022-00563-0 PMC9618413

[10] Tan TG, Mui E, Cong H, Witola WH, Montpetit A, Muench SP, et al. Identification of T. gondii epitopes, adjuvants, and host genetic factors that influence protection of mice and humans. Vaccine. 2010;28(23):3977–89. 20347630 10.1016/j.vaccine.2010.03.028 PMC2895808

[11] Skwarczynski M, Zaman M, Urbani CN, Lin I, Jia Z, Batzloff MR, et al. Polyacrylate dendrimer nanoparticles: a self-adjuvanting vaccine delivery system. Angew Chem Int Ed. 2010;49(33):5742–5. 10.1002/anie.201002221 20818757

[12] Sola F, Canonico B, Montanari M, Volpe A, Barattini C, Pellegrino C, et al. Uptake and intracellular trafficking studies of multiple dye-doped core-shell silica nanoparticles in lymphoid and myeloid cells. NSA. 2021;14:29–48. 10.2147/NSA.S290867 PMC7954439 33727804

[13] Fang RH, Zhang L. Nanoparticle-based modulation of the immune system. Annu Rev Chem Biomol Eng. 2016;7(1):305–26. 27146556 10.1146/annurev-chembioeng-080615-034446

[14] Dacoba TG, Olivera A, Torres D, Crecente-Campo J, Alonso MJ. Modulating the immune system through nanotechnology. Semin Immunol. 2017;34:78–102. 29032891 10.1016/j.smim.2017.09.007 PMC5774666

[15] Lê MQ, Carpentier R, Lantier I, Ducournau C, Fasquelle F, Dimier-Poisson I, et al. Protein delivery by porous cationic maltodextrin-based nanoparticles into nasal mucosal cells: Comparison with cationic or anionic nanoparticles. Int J Pharm X. 2019;1:100001. 31545856 10.1016/j.ijpx.2018.100001 PMC6733295

[16] Bernocchi B, Carpentier R, Lantier I, Ducournau C, Dimier-Poisson I, Betbeder D. Mechanisms allowing protein delivery in nasal mucosa using NPL nanoparticles. J Control Rel. 2016;232:42–50. 10.1016/j.jconrel.2016.04.014 PMC4907310 27080572

[17] Fasquelle F, Carpentier R, Demouveaux B, Desseyn JL, Betbeder D. Importance of the Phospholipid Core for Mucin Hydrogel Penetration and Mucosal Cell Uptake of Maltodextrin Nanoparticles. ACS Applied Bio Materials. 2020;3(9):5741–9. 10.1021/acsabm.0c00521. 10.1021/acsabm.0c00521 35021805

[18] Carpentier R, Platel A, Salah N, Nesslany F, Betbeder D. Porous maltodextrin-based nanoparticles: a safe delivery system for nasal vaccines. J Nanomater. 2018;2018:1–8.

[19] Fasquelle F, Dubuquoy L, Betbeder D. Starch-based NP act as antigen delivery systems without immunomodulating effect. PLoS ONE. 2022;17(7):0272234 Omri A, editor. 10.1371/journal.pone.0272234 PMC9337643 35905121

[20] Ducournau C, Cantin P, Alerte V, Quintard B, Popelin-Wedlarski F, Wedlarski R, et al. Vaccination of squirrel monkeys (Saimiri spp.) with nanoparticle-based Toxoplasma gondii antigens: new hope for captive susceptible species. Int J Parasitol. 2023;53(7):333–46. 36997082 10.1016/j.ijpara.2023.02.003

[21] Ducournau C, Moiré N, Carpentier R, Cantin P, Herkt C, Lantier I, et al. Effective nanoparticle-based nasal vaccine against latent and congenital toxoplasmosis in sheep. Front Immunol. 2020;11(September):1–13. 33013917 10.3389/fimmu.2020.02183 PMC7509486

[22] Ducournau C, Nguyen TT, Carpentier R, Lantier I, Germon S, Précausta F, et al. Synthetic parasites: a successful mucosal nanoparticle vaccine against Toxoplasma congenital infection in mice. Future Microbiol. 2017;12(5):393–405. 28339296 10.2217/fmb-2016-0146

[23] Fasquelle F, Scuotto A, Vreulx AC, Petit T, Charpentier T, Betbeder D. Nasal vaccination of six squirrel monkeys (Saimiri sciureus): Improved immunization protocol against Toxoplasma gondii with a nanoparticle-born vaccine. Int J Parasitol: Parasites Wildlife. 2023;22:69–74. 10.1016/j.ijppaw.2023.09.002 PMC10500419 37720360

[24] Dimier-Poisson I, Carpentier R, N’Guyen TTL, Dahmani F, Ducournau C, Betbeder D. Porous nanoparticles as delivery system of complex antigens for an effective vaccine against acute and chronic Toxoplasma gondii infection. Biomaterials. 2015;50:164–75. 25736506 10.1016/j.biomaterials.2015.01.056

[25] Rosowski EE, Nguyen QP, Camejo A, Spooner E, Saeij JPJ. Toxoplasma gondii inhibits gamma interferon (IFN-γ)- and IFN-β-induced host cell STAT1 transcriptional activity by increasing the association of STAT1 with DNA. Infect Immun. 2014;82(2):706–19 Adams JH, editor. 24478085 10.1128/IAI.01291-13 PMC3911376

[26] Ivanova DL, Krempels R, Denton SL, Fettel KD, Saltz GM, Rach D, et al. NK cells negatively regulate CD8 T cells to promote immune exhaustion and chronic toxoplasma gondii infection. Front Cell Infect Microbiol. 2020;10:313. 32733814 10.3389/fcimb.2020.00313 PMC7360721

[27] Sa Q, Tiwari A, Ochiai E, Mullins J, Suzuki Y. Inducible nitric oxide synthase in innate immune cells is important for restricting cyst formation of Toxoplasma gondii in the brain but not required for the protective immune process to remove the cysts. Microbes Infect. 2018;20(4):261–6. 29287983 10.1016/j.micinf.2017.12.004 PMC5911423

[28] Holley CK, Cedrone E, Donohue D, Neun BW, Verthelyi D, Pang ES, et al. An in vitro assessment of immunostimulatory responses to ten model Innate Immune Response Modulating Impurities (IIRMIs) and peptide drug product, teriparatide. Molecules. 2021;26(24):7461. 34946542 10.3390/molecules26247461 PMC8707785

[29] González PA, Carreño LJ, Coombs D, Mora JE, Palmieri E, Goldstein B, et al. T cell receptor binding kinetics required for T cell activation depend on the density of cognate ligand on the antigen-presenting cell. Proc Natl Acad Sci USA. 2005;102(13):4824–9. 15772168 10.1073/pnas.0500922102 PMC555720

[30] Gómez Marín JE, Bonhomme A, Guenounou M, Pinon JM. Role of interferon-γ against invasion by Toxoplasma gondii in a human monocytic cell line (THP1): Involvement of the parasite’s secretory phospholipase A2. Cell Immunol. 1996;169(2):218–25. 8620549 10.1006/cimm.1996.0112

[31] Gomez-Marín JE, El’Btaouri H, Bonhomme A, Antonicelli F, Pezzella N, Burlet H, et al. Involvement of secretory and cytosolic phospholipases a2 during infection of IHP1 human monecytic cells with Toxoplasma gondii. Effect of interferon γ. Parasitol Res. 2002;88(3):208–16. 11954905 10.1007/s00436-001-0525-z

