# Supplementary figures and images for: T-cell activation of Toxoplasma gondii positive donors by maltodextrin nanoparticles formulated with killed Toxoplasma gondii
Source: BMC Infect Dis. 2025 Feb 26;25:279. doi: 10.1186/s12879-025-10656-5 (PMC11866635; doi:10.1186/s12879-025-10656-5)

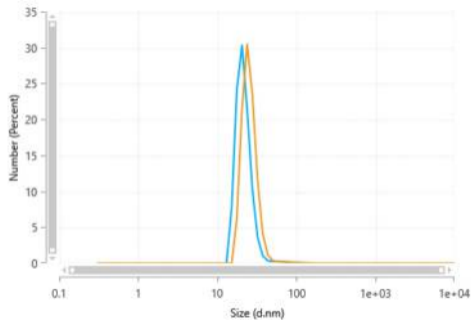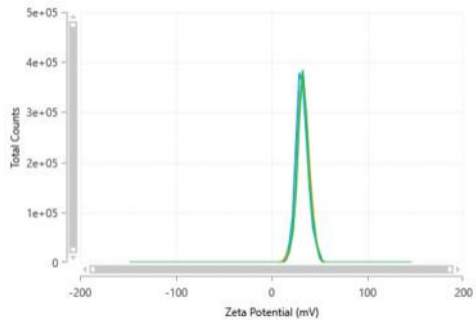

Supplement: Supplementary file 1 — Additional file 1. Characterization of sizeand surface chargeof empty NPL before the antigen encapsulation [file 12879_2025_10656_MOESM1_ESM.pdf]

## % Population in PBMC

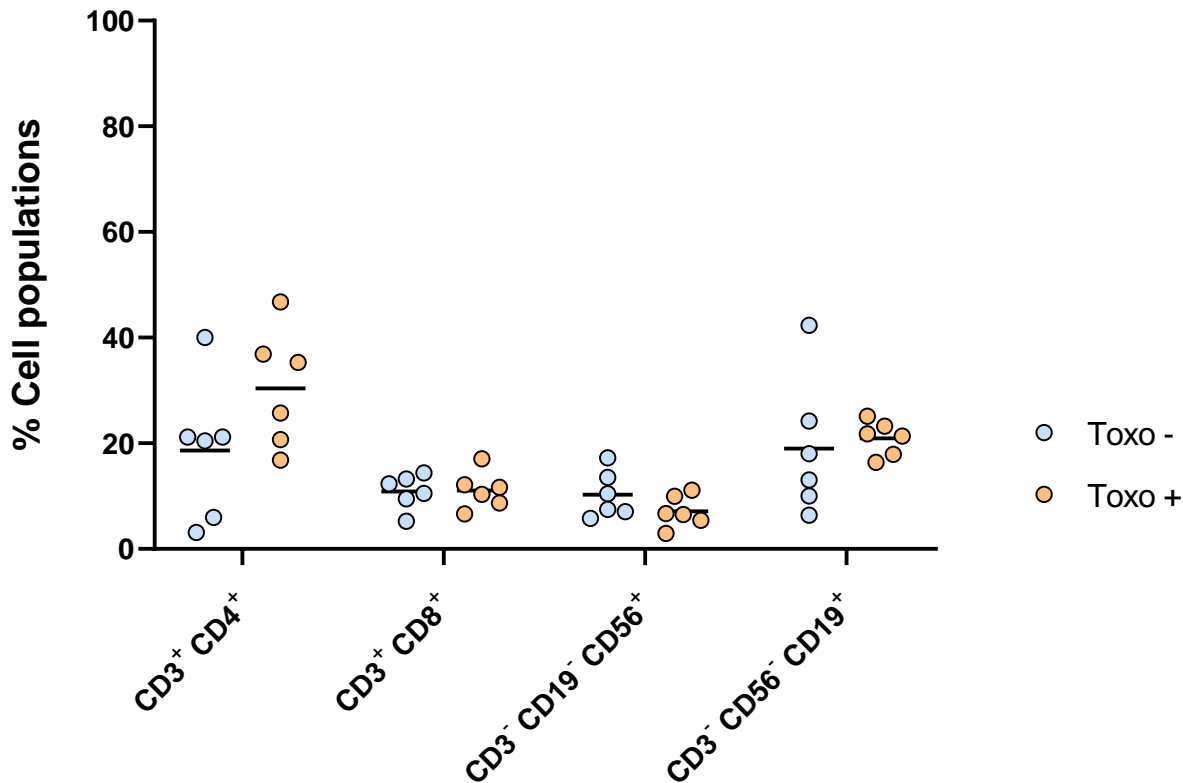

Supplement: Supplementary file 2 — Additional file 2. Frequencies of PBMC subsetsbetween Toxo – and Toxo + donors, measured by flow cytometry. [file 12879_2025_10656_MOESM2_ESM.pdf]
